# Reconciling *in vivo* and *in silico* key biological parameters of *Pseudomonas putida* KT2440 during growth on glucose under carbon-limited condition

**DOI:** 10.1186/1472-6750-13-93

**Published:** 2013-10-29

**Authors:** Jozef BJH van Duuren, Jacek Puchałka, Astrid E Mars, René Bücker, Gerrit Eggink, Christoph Wittmann, Vítor AP Martins dos Santos

**Affiliations:** 1 Systems and Synthetic Biology Group, Helmholtz Centre for Infection Research, Inhoffenstraße 7, D-38124, Braunschweig, Germany; 2 Wageningen UR Food&Biobased Research, P.O. Box 17, 6700 AA Wageningen, The Netherlands; 3 Bioprocess Engineering Group, Wageningen University, P.O. Box 8129, 6700, EV Wageningen, The Netherlands; 4 Institute of Biochemical Engineering, Technische Universität Braunschweig, Gauβstraβe 17, D-38106 Braunschweig, Germany; 5 Kluyver Centre for Genomics of Industrial Fermentation, P.O. Box 5057, 2600, GA Delft, The Netherlands; 6 LifeGlimmer GmbH, Markelstraße 39a, D-12163 Berlin, Germany; 7 Present address: Dr. von Hauner Children’s Hospital, Ludwig-Maximilians-University Munich, Lindwurmstraße 4, D-80337 München, Germany; 8 Present address: Laboratory of Systems and Synthetic Biology, Wageningen University, P.O. Box 8033, 6700, EJ Wageningen, The Netherlands

**Keywords:** Continuous cultivation, *P. putida* KT2440, Glucose, Metabolic modeling, Biomass composition, Transcriptomics

## Abstract

**Background:**

Genome scale metabolic reconstructions are developed to efficiently engineer biocatalysts and bioprocesses based on a rational approach. However, in most reconstructions, due to the lack of appropriate measurements, experimentally determined growth parameters are simply taken from literature including other organisms, which reduces the usefulness and suitability of these models. *Pseudomonas putida* KT2440 is an outstanding biocatalyst given its versatile metabolism, its ability to generate sufficient energy and turnover of NADH and NAD. To apply this strain optimally in industrial production, a previously developed genome-scale metabolic model (iJP815) was experimentally assessed and streamlined to enable accurate predictions of the outcome of metabolic engineering approaches.

**Results:**

To substantially improve the accuracy of the genome scale model (iJP815), continuous bioreactor cultures on a mineral medium with glucose as a sole carbon source were carried out at different dilution rates, which covered pulling analysis of the macromolecular composition of the biomass. Besides, the maximum biomass yield (on substrate) of 0.397 g_DCW_ · g_glc_^-1^, the maintenance coefficient of 0.037 g_glc_ · g_DCW_^-1^ · h^-1^ and the maximum specific growth rate of 0.59 h^-1^ were determined. Only the DNA fraction increased with the specific growth rate. This resulted in reliable estimation for the Growth-Associated Maintenance (GAM) of 85 mmol_ATP_ · g_DCW_^-1^ and the Non Growth-Associated Maintenance (NGAM) of 3.96 mmol_ATP_ · g_DCW_^-1^ · h^-1^. Both values were found significantly different from previous assignment as a consequence of a lower yield and higher maintenance coefficient than originally assumed. Contrasting already published ^13^C flux measurements and the improved model allowed for constraining the solution space, by eliminating futile cycles. Furthermore, the model predictions were compared with transcriptomic data at overall good consistency, which helped to identify missing links.

**Conclusions:**

By careful interpretation of growth stoichiometry and kinetics when grown in the presence of glucose, this work reports on an accurate genome scale metabolic model of *Pseudomonas putida*, providing a solid basis for its use in designing superior strains for biocatalysis. By consideration of substrate specific variation in stoichiometry and kinetics, it can be extended to other substrates and new mutants.

## Background

*Pseudomonas putida* is a non-pathogenic, soil bacterium that developed a notably versatile metabolism and thus can inhabit several environmental niches. Numerous strains, some of them being solvent tolerant [1,2], are able to produce efficiently a range of bulk and fine chemicals or, conversely, degrade various substances that are by products or waste of industrial processes [3-5]. These features, along with the amenability for genetic manipulation and suitability as a host for the expression of heterologous genes have rendered *P. putida* an attractive object of research for biotechnological applications [6].

The sequencing of the genome of *P. putida* brought a significant leap in developing its applications by unveiling the metabolic potential encoded in the genome [7]. In an effort to enable the analysis of *P. putida* KT2440 from a systems biology perspective and to foster the development of its biotechnological applications, we built a constraint-based metabolic reconstruction (iJP815) of this strain [8,9]. Although a lot of knowledge pertaining to this strain was collected while building the model, some information had to be approximated from the *Escherichia coli* model [10].

The information approximated included the biomass composition of the bacterium and the energetic expenses related to growth and maintenance of life-functions (the so called Growth Associated coefficient and Non-Growth Associated Maintenance coefficient (GAM and NGAM)) [11]. One of the most common assumptions underlying simulations built upon genome-scale constraint-based metabolic reconstructions is that bacteria try to maximize their growth yield. Consequently, growth associated parameters can be specified prior to performing simulations of the growth, which provide a basis for numerous applications of metabolic reconstructions including rational strain design for biotechnological (and other) applications. Furthermore, these parameters influence significantly the outcome of such simulations, as they confine fluxes of important parts of the metabolism [12-15]. Moreover, they may vary significantly not only among organisms, but also among different growth conditions. Thus, for applications of metabolic reconstructions in biotechnology, the knowledge of maintenance coefficients is pivotal as their coefficients determine how much of the supply is “wasted” for the activities not related to the investigated application itself.

To increase the accuracy of the genome-scale metabolic model of *P. putida* (iJP815) growth related parameters as well as the macro molecular composition of the biomass were measured carefully during continuous bioreactor cultures at different dilution rates with glucose as sole carbon limited source. The solution space of the improved model was constrained, based on the newly measured growth parameters and by contrasting model simulations with available ^13^C flux measurements [16]. The augmented accuracy led to an increased flux for the acetyl-CoA production, which anti correlated strongly with the change of biomass yield (*Y*_*x/s*_). Moreover, transcriptomic data with good consistency with the model predictions were used to find inconsistencies within the model by comparing expression levels with simulations [17].

## Results

### Macromolecular components

**Figure 1:**
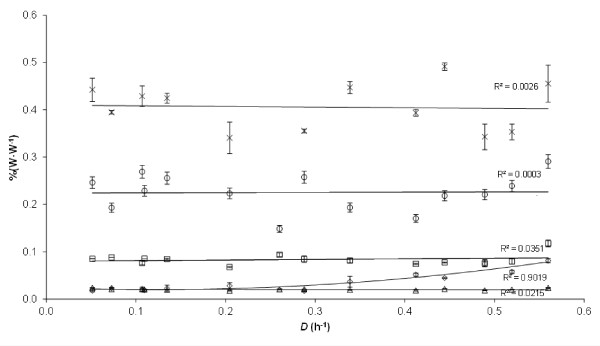
**Macromolecular composition of *P. putida *KT2440 grown in continuous culture on glucose at different dilution rates ( *D *) (h**^**-1**^**).** The percentages of water-soluble proteins (×), lipids (□), carbohydrates (Δ), RNA (○), and DNA (◊) in the total biomass in % (W · W^-1^) are given.

The molecular composition of the *P. putida* KT2440 cell remained fairly stable over the whole measured range of *D*. The biomass fractions of RNA, lipids, water-soluble proteins, and carbohydrates showed no significant dependency on the μ, based on linear regression (Figure 1. R^2^ shown), whereas DNA exhibited an increase (quadratic regression, p-value: 9.0E-6). The standard deviation of these measurements is based on the variability of optical density (OD) measurements. The macromolecular composition of the biomass is summarized in Table 1. About 90.3% (±7.9%) of the dry cell weight (*C*_*x*_) was identified with the measurements of the macromolecules. The mass missing in the macromolecular measurements justified the addition of 12.2% hydrophobic proteins [18,19]. Water-insoluble proteins, peptidoglycan, vitamins and co-factors were not included in the measurements. By balancing carbon and nitrogen measurements of the continuous fermentations, the average fraction of these compounds in the *C*_*x*_ was computed to be 52.5% (±2.5) and 14.3% (±1.4), respectively. The amino acid composition of the proteins of the cells grown at a dilution rate (*D*) of 0.2 h^-1^ was analyzed (Table 2). By dividing the total concentration of amino acids by the measured *C*_*x*_ the mass of proteins constituted 49.7% (±0.05%) of the biomass. When the none-identified amino acid concentrations are included of cysteine, methionine, and tryptophan, based on the molar fractions as in *E. coli*[10], the final protein concentration amounts 55.3%. This concentration seems to be very similar to the total protein concentration of the original model (iJP815) at 55.3% (W·W^-1^) and the total protein concentration determined at 52.8 (±1.2)% (W·W^-1^).

**Table 1:** **The comparison of macromolecular composition of the *P. putida *** **KT2440 biomass**

**Macro-molecule**	**% (W · W**^**-1**^**) *P. putida *** **KT2440 ± standard deviation (this study)**	**% (W · W**^**-1**^**) iJP815 ( *E. coli *) biomass**[8]	**% (W · W**^**-1**^**) PpuMBEL1071 *P. putida *** **KT2440**[20]
Proteins	52.8 ± 1.2^*^	55.3^*^	50.6^*^
Lipids	8.4 ± 0.3	13.0	7.4^***^
Carbohydrates	2.1 ± 0.1	2.5	
RNA	22.6 ± 0.0	20.7	20
DNA	2.2 ± 0.3^**^	3.1	2.8

(^*^Pertains to all proteins, ^**^D of 0,2, ^***^phospholipids and lipopolysaccharide).

**Table 2:** **Amino acid composition of the proteins of *P. putida *** **KT2440 at a dilution rate ( *D *) of 0.2 h**^**-1**^

**Amino acid**	**Mol % (this study)**	**Mol % PpuMBEL1071**[20]
Ala	12	10
Arg	5	6
Asx	10	9
Cys		2
Glx	12	10
Gly	10	12
His	2	2
Ile	4	5
Leu	10	8
Lys	5	6
Met		3
Phe	4	4
Pro	5	4
Ser	5	4
Thr	5	5
Trp		1
Tyr	3	3
Val	7	8

Concentrations of cysteine, methionine, and tryptophan could not be identified in this assay and were assumed to have identical molar fractions as in the *E. coli* biomass [10].

Asx = Asp + Asn; Glx = Glm + Gln.

### Creation of *P. putida* specific biomass equation

A new biomass equation for the metabolic model (iJP815) was computed based on the cell composition at a *D* of 0.2 h^-1^ and other sources (see Methods). This new equation differed only to some extent from the old one, with respect to both macromolecular and elemental composition (Tables 1 and 3). The main difference for the macromolecular composition is the lower fraction of lipids and the higher fraction of RNA (see Table 1). From the *in silico* biomass equation created in this work and from the *in silico* biomass equation used in the original model iJP815 model (*E. coli* biomass) [10], the elemental composition of *P. putida* KT2440 was computed, which is shown in Table 3. This composition corresponded well with the elemental compositions that were calculated from the C and N balances and the values that were reported for the ashing and chemical determination of the biomass from *P. putida* ATCC 29735 grown in continuous culture on peptone-yeast extract medium [21]. The lower carbon fraction in the *in silico* biomass and the appropriate nitrogen concentration could suggest that the lipids fraction was underestimated since this macromolecule has a carbon fraction exceeding 50% and contains a relative low concentration of nitrogen [13].

**Table 3:** **Elemental composition of the biomass % (W · W**^**-1**^**)**

	**Calculated from *in silico P. putida *** **specific biomass (this study)**	**C-/N-balance (this study)**	**Ashing and chemical preparation**[21]	**Calculated from *in silico *** **original iJP815 ( *E. coli *) biomass**
C	48.8	52.5	52.1	49.6
H	6.2		7.4	6.4
N	15.2	14.3	14.3	14.8
O	26.4			25.6
P	2.7		1.8	2.8
S	0.7		0.5	0.7

In order to assess the effect of change of the biomass equation on the accuracy of predicted flux values, the flux variability analysis (FVA)-distances were computed from simulations performed using either biomass equation. The maximal and minimal FVA-distances changed from 2229 and 72 to 2228 and 71, respectively. As a result the new biomass equation caused no significant change of the FVA-distances, which is in agreement with the small differences between the biomass equations.

### Growth related factors

**Figure 2:**
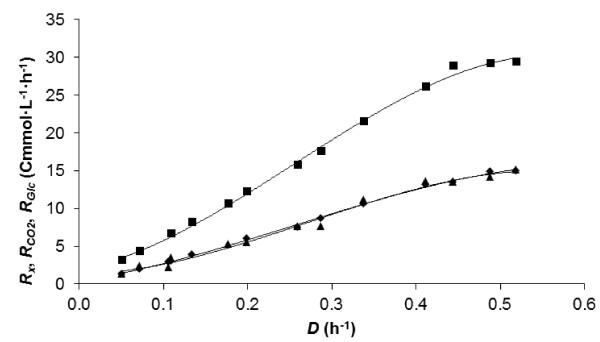
**The biomass production rate (*R***_***x***_**) (♦), CO2 production rate (*R***_***CO2***_**) (▲), glucose uptake rate (*R***_***glc***_**) (■) (Cmmol∙L**^**-1**^ **· h**^**-1**^**) of *P. putida *KT2440 at various dilution rates (*D*) (h**^**-1**^**) on MM with 10 mM glucose.**

**Figure 3:**
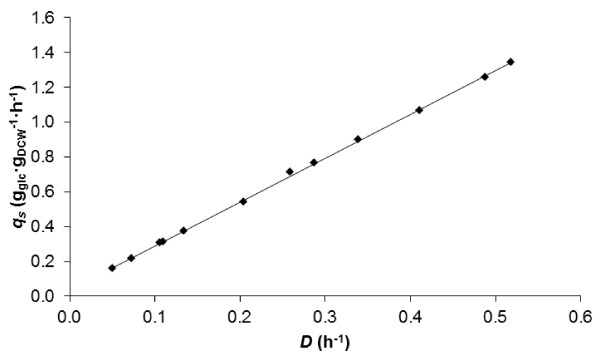
**The specific rate of substrate utilization (*q***_***s***_**) (g**_**glc**_ **· g**_**DCW**_^**-1**^ **· h**^**-1**^**) at various dilution rates (*D*) (h**^**-1**^**) on MM with 10 mM glucose (Pirt diagram)**[23]**.**

Not only biomass composition, but also growth-related parameters were determined during the continuous fermentations of *P. putida* KT2440 on glucose at different *D*. The biomass production rate (*R*_*x*_), CO_2_ production rate (*R*_*CO2*_), and glucose uptake rate (*R*_*glc*_) increased until *D* 0.49 h^-1^ (Figure 2) with the highest measured *Y*_*x/s*_ of 0.389 g_DCW_ · g_glc_^-1^. The maximum growth rate (*μ*_*max*_) was determined to be 0.59 h^-1^ by a wash out experiment (r^2^ of 0.98). This means that when *P. putida* KT2440 was cultivated with the highest *D* of 0.64 h^-1^ the specific growth rate (*μ*) was equal to *μ*_*max*_ and as a consequence no steady state was reached. Above *D* of 0.52 h^-1^ an increasing glucose concentration (*C*_*glc*_) was observed (see Additional file 1: Figure S1). This is most probably a consequence of approaching the *μ*_*max*_, whereas the classical Monod-relationship [22] was not observed (the relationship between *C*_*glc*_ and *D* (*μ*_*max*_-*D*)^-1^ was exponential rather than linear). The maximal biomass yield (*Y*_*x/s*_^*max*^) and the maintenance coefficient (*m*_*s*_) of *P. putida* KT2440 were determined with high accuracy to be 0.397 g_DCW_ · g_glc_^-1^ and 0.037 g_glc_ · g_DCW_^-1^ · h^-1^, respectively (Figure 3). The linear regression was very strong, as r^2^ = 1.0.

The average concentration of cells was 7.19E + 08 (±14%) cells per mL. The trend was decreasing with an increasing *D*. On average 95 (±3)% of the cells were alive (*C*_*x*_*live*) and 2 (±2)% were dead (*C*_*x*_*dead*). Furthermore, the increase of *Y*_*X/S*_ at higher *D* is caused by a gradual increase of innercomplexity (side scatter) and an abrupt augmentation of the cell size at a *μ* of 0.4 h^-1^ (forward scatter). This is illustrated by a distinctive trend of forward and side scatter plots, measured by FACSCalibur flow cytometer (BD Biosciences, USA) (see Additional file 2: Figure S2). The analysis of feed medium by nuclear magnetic resource (NMR) showed strong characteristic signals of the α- and β-anomeric forms of D-glucopyranose together with those of the antifoam, as well as very small signals of impurities associated with the technical grade materials used (data not shown). During steady state of the fermentation the signals for glucose had completely disappeared and only antifoam and those of the small impurity signals could be detected. The signal-to-noise ratio was high enough to show that no organic metabolites as e.g. acetate, proteins or peptides accumulated during the fermentation. The position of such signals was in regions of the spectrum that were essentially free from overlap. Because no other metabolites were found in the medium by ^1^H-NMR at a *D* of 0.26, 0.44 and 0.49 h^-1^, it was concluded that there is no accumulation of any intermediates of anabolism or catabolism in the range of *D* under carbon limited conditions.

### New maintenance coefficients

Based on the experimental data the *Y*_*x/s*_^*max*^ and *m*_*s*_ were estimated, and used to fit the growth associated maintenance (GAM) and the non-growth associated maintenance (NGAM) parameters of iJP815. These were computed to be 85 mmol_ATP_ g_DCW_^-1^ and 3.96 mmol_ATP_ g_DCW_^-1^ · h^-1^ for GAM and NGAM, respectively. Due to a lower *Y*_*x/s*_^*max*^ than assumed in the original model the GAM increased. The determined *Y*_*x/s*_^*max*^ is 22 and 25 percent lower than in the original *P. putida* KT2440 and *E. coli* models, respectively. The NGAM was decreased approximately [10,11] as a consequence of lower value of the *m*_*s*_. The change of GAM and NGAM coefficients caused larger changes to the flux distribution than the change of biomass equation. The min FVA-distance did decrease by 8% and the maximal FVA-coefficient did manifest itself unexpectedly by an increased maximal FVA-distance of 10%. However the numbers were still in the same range.

### Evaluation of limits within the central metabolism

When inspecting the FVA-results for the reactions whose fluxes were derived via ^13^C-measurements [16], one enzyme was identified that showed a high possible variability of activity, namely malate dehydrogenase (EC:1.1.1.38). It was next tested, how the restriction of the flux of the respective reaction, during growth on glucose, would influence the output of FVA-simulations. The reaction was constrained to the experimentally measured flux value based on ^13^C measurement. Imposing this limit, had a very strong impact on the flux prediction. The drop in the FVA-distance was much higher than implied by the limit on the particular reaction. This drop was mainly observed for the maximal distance, leading to a residue of 3% of the original solution space. The minimal solution space was constrained for 10%. As a consequence, constraining this reaction did partly lead to identification of a solution that is closer to the experimental value by significantly minimizing the minimal distance in the original model, but it mainly excluded futile cycles that are far from the experimental. The FVA predicts in such a case, infinite fluxes. The limitation of malate dehydrogenase led to a minimal and maximal FVA-distance of 61 and 65, which are in the same range of values.

Isotopic ^13^C measurements showed that glucose 6-phosphate isomerase (EC: 5.3.1.9) operated in the reverse direction. FVA could not determine such inefficiency, given it is not in line with the evolutionary selection to reach a high *Y*_*x/s*_^max^ as defined by the function of the model.

### Influence of growth stoichiometry and maintenance changes on model predictions

The original *P. putida* model was used to generate hypotheses pertaining to increasing the production of acetyl-CoA [8]. This compound is an entry metabolite for the synthesis of polyhydroxyalkanoates – biodegradable plastics of industrial and medical relevance [24,25]. Mutants described by Poblete Castro *et al*. showed an increased Acetyl-CoA production flux [26]. In order to evaluate, how the changes of biomass composition and maintenance coefficients influenced predictions of the model, the FVA-calculation for the previously defined mutant with blocked enzymatic activity for glucose dehydrogenase (PP1444) was executed for the old and new conditions without any constraints. Compared to the old values, the new values lead to an decrease in the maximum AcCoA production flux and *Y*_*x/s*_^*max*^: from 25.6 to 21.2 [mmol·g_DCW_·h^-1^] and from 0.34 to 0.32 [g_DCW_·g_glc_^-1^], respectively, which thus narrows down the predictive range.

### Evaluating the consistency between the transcriptomic data and the model

To further validate the model, the consistency between model predictions and the transcriptomics data, generated at steady-state, was assessed. The flux balance analysis (FBA)-predicted fluxes and the levels of respective mRNA transcripts showed overall good agreement with each other. It should be emphasized, however, that measured abundances of mRNA transcripts cannot be directly translated into fluxes. The consistency was evaluated by analyzing the expression levels of: i) genes essential for growth, ii) non-essential genes with low expression that are involved in reactions with non-zero flux, and iii) genes with high expression values that are only involved in the catalysis of the reactions with predicted zero flux.

Expression of the genes essential for growth: The first assessment aimed at identifying genes essential for growth *in silico* that are actually not expressed. From genes identified by the model as essential those were selected and termed as potentially not expressed that complied with at least one of the following criteria: i) the number of “absent” calls (as defined by Affymetrix analysis Manual) was higher than 2 (out of 4 arrays), or ii) the number of “present” calls was lower than 3, (out of 4 arrays) or iii) the mean log_2_ absolute gene expression value was lower than 7.5 ( threshold, below which genes do not seem to be expressed). Out of 147 essential genes 33 were selected that were potentially not expressed. They are listed (see Additional file 3: Table S1). Inspection of these genes showed that the overlap between the methods is limited. The “present” call condition is the least restrictive one, while the mean condition is the most restrictive. Many of the genes that fulfilled one of the “call” conditions show log_2_ absolute gene expression values higher than 8, whereas the average over all arrays was 8.29. This suggests that the call methods are relatively untrustworthy and that, in many cases, it rather points out the genes for which experimental problems possibly occurred, e.g. due to cross-hybridization of the respective probes with mRNA from some other gene. A cluster of genes PP0237-PP0240 (rows marked blue) that was originally annotated to be involved in sulfate transport was consistently identified as absent. After close inspection, these genes appeared to be miss-assigned, since there was a group of other genes that is responsible for sulfate transport (PP5168-PP5171). The model was updated accordingly.

Inspecting the genes that complied with at least two of the criteria revealed that there were a number of those involved in biosynthesis of cofactors (red rows). The corresponding reactions usually have a low flux so it may be that low protein levels suffice for the required enzymatic activity. There are also six genes involved in the synthesis of LPS (green). The majority of these genes has relatively high expression levels and were selected due to the presence/absence criteria. LPS is needed in rather limited amounts so a low expression may be sufficient for the required enzymatic activity. Furthermore these six genes constitute only around 50% of the genes involved in LPS synthesis, so this also suggests that the expression levels are sufficient. PP2458, PP2460, and PP4266 (yellow) are genes that were labeled as essential due incompleteness of the metabolic model. The reason these genes are essential is, that S-adenosyl homocysteine is produced during the synthesis of cyclo-fatty acids. This compound needs to be converted further. Apparently the only way is to convert it into ribose and adenine which are then phosphorylated to ribose-6-phosphate and AMP, respectively. This route seems, however, not to be the proper one.

Expression of non-essential genes required for optimal growth: The second assessment was an extension to the previous one. Here reactions with non-zero minimal fluxes in FVA-simulations were identified. For these reactions the “expression value” was computed and reactions with a log_2_ expression value below 7.5 were selected. There were only five such reactions identified. They were all involved in fatty acid biosynthesis and all catalyzed by the same pair of isozymes (PP3553 and PP2795-acyl-CoA synthetases) whose mean expression values did both not exceed 7.5.

Highly expressed genes not required for growth: Finally, genes with high expression levels were identified that only catalyze reactions with a low flux (this threshold was arbitrarily set to 0.1 mmol∙g_DCW_^-1^∙h^-1^, when the *μ* was 0.2 h^-1^). These genes are listed (see Additional file 4: Table S2). Several interesting observations could be made out of it. First, branched-chain amino acids ABC transporters constituted a significant fraction of these genes (blue rows), suggesting that the bacterium is actively searching for alternative nutrition sources. Second, a number of genes are involved in the synthesis of cofactors as pyridoxal phosphate (CAS number 54-47-7) and vitamins which include biotin (CAS number 58-85-5), thiamin pyrophosphate (CAS number 154-87-0), and riboflavin (CAS number 83-88-5) (yellow rows). For the last one, only a single gene from its biosynthesis pathway showed high expression and, as riboflavin is included into biomass composition, there is a flux going through the reactions involved in its synthesis. This flux depends on the share of the riboflavin in the biomass. The high expression of this gene may suggest that this coefficient should be higher, yet the evidence is weak, as other genes belonging to the pathway did not show high expression. The other three compounds are not included in the biomass equation, but the expression levels of respective genes suggest that they should be included. The mechanism (biosynthetic reactions and genes catalyzing them) of the synthesis of pyridoxal phosphate in *P. putida* KT2440 is known, whereas it is not the case for the other two compounds. Third, reactions involved in rhamnosugars synthesis (red rows) are also highly expressed. These are used for synthesis of rhamnolipids that are a constituent of the bacterial envelope and are not part of the biomass in the current version of the model. This assay suggests that they should be included. This requires however the determination of the exact composition of these metabolites and their share in the biomass. The reason of higher expression of remaining genes could not be elucidated.

## Discussion

Constraint-based modeling provides a valuable framework for navigating microbial metabolic networks and for identification and prediction of intra-cellular flux distributions, thereby aiding the discovery and application of bacteria e.g. in biotechnology [27]. However, to be reasonably predictive, the metabolic reconstructions created within this framework require proper and accurate input information. Striving to improve the metabolic reconstruction of *P. putida* KT2440, we characterized experimentally the macromolecular composition of the bacterium cultivated continuously on a minimal medium with glucose as the sole carbon and energy source at a range of *D* (0.05-0.6 h^-1^). These measurements showed that the macromolecular biomass composition does not change significantly when the *D* and thus the *μ* varies. The only macromolecule that did change significantly upon increasing *D* was the DNA. This might have been a consequence of the appearance of a higher number of replication forks. At a *μ* of 0.4 h^-1^ the cell size increases rapidly, which might be related to the increase of genome replication [28]. In other organisms like *E. coli* K12 (*D* range: 0.05-1.7 h^-1^) [12], *E. coli* B/r (*D* range: 0.6-2.5 h^-1^) [28], and *P. denitrificans* (*D* range: 0.06-0.6 h^-1^) [12], the DNA fraction was observed to decrease at increasing *D*, or remain stable as in *S. cerevisiae* (*D* range: 0.02-0.2 h^-1^) [13] and *A. calcoaceticus* (*D* range: 0.01-0.95 h^-1^) [29]. Baart *et al.*[30] observed however that in *N. meningitidis* (*D* range: 0.04-0.16 h^-1^) the DNA fraction increases too. The stability of the RNA and protein fractions in *P. putida* KT2440 also contradicts to some extent previous findings, as in some other organisms – *E. coli* and *P. denitrificans* – these fractions increased and decreased with the *D,* respectively [12,28]. However, no significant changes were observed for the RNA and protein fractions in *E. coli* K12 at the *D* range of 0.05-0.6 h^-1^ and *N. meningitides* at the *D* range of 0.04-0.16 h^-1^[12,30]. In *A. calcoaceticus* (*D* range: 0.01-0.95 h^-1^) [29] only the RNA fraction increased and in *S. cerevisiae* (*D* range: 0.02-0.2 h^-1^) [13] both the RNA and protein fractions increased. The increase of the RNA fraction most probably reflects the extra need for ribosomal RNA for the synthesis of proteins [31]. However, besides the increase of the number of ribosomes, the higher need for proteins can be fulfilled by an increase in the efficiency of their synthesis [29].

Based on these results and a multitude of different sources, including our amino acid composition measurements, a new *P. putida* specific biomass equation was created. The differences between the new and old biomass equation were relatively small, showing that the biomass compositions of *P. putida* and *E. coli* do not differ significantly. This supports the assumption made in many reconstruction efforts that *E. coli* biomass composition approximates well that of other gram-negative bacteria when grown at the same *D*. The protein composition is comparable to that determined by Sohn *et al*. [20]. On the contrary, the peptidoglycan concentration reached 22.7% of the biomass weight in the macro molecular biomass composition. This concentration was surprisingly high as the sum of the fractions exceeded 100%. Of the determined growth related factors, the specific rate of substrate utilization (*q*_*s*_) increased linearly with the *D* when the culture was glucose-limited. This is in agreement with the *Y*_*x/s*_/*m*_*s*_ model of Pirt [23]. *Y*_*x/s*_^*max*^ and *m*_*s*_ were used to update the coefficients of GAM and NGAM in the model. In contrast to the biomass composition, the new values differed substantially from the old ones. Particularly the *Y*_*x/s*_^*max*^ was significantly lower and, consequently, the GAM higher, than assumed before. This means that the energy required for assembly of a cell of *P. putida* is much higher than in *E. coli*, which GAM coefficient was used in the original version of the model. This resulted in less energy being available for biotechnological processes. On the other hand even at a *D* of 0.49 h^-1^, no overflow metabolism was observed, i.e. no by-products (e.g. acetate) were accumulated. The opposite is the case for many organisms at high *μ* values and is a result of limitations in the TCA cycle or respiratory NADH turnover [12,32,33]. For example, in *E. coli* cultures at *D* above 0.4 h^-1^ acetate accumulates in the medium [32]. The lack of overflow metabolism is of great importance as it points out the capacity of *P. putida* KT2440 as biocatalyst. The ability to generate sufficient energy and the availability of NADH and NAD + are of importance for applications of a strain in biocatalysis, especially for a cofactor dependent conversion and the primary export of the product out of the cell. These results confirm earlier presented data that *P. putida* can meet high energy demands [34,35]. Controlling the availability of the energy equivalent ATP at a sufficient level during the production phase, forms an interesting lead to optimize the biocatalyst by using metabolic engineering. After implementing the new biomass equation and the new coefficients of GAM and NGAM the model was contrasted with ^13^C-flux measurement (used also for the validation of the original model). The new biomass, GAM, and NGAM did not largely influence the range of the distances between FVA-solutions. The increased maximum FVA-distance after implementing the new GAM and NGAM can possibly be explained by the fixed glucose uptake instead of additional conversion of glucose to gluconate and 2-ketogluconate. If we look at the similar FVA-distances it may mean that the model itself was not improved (because a good agreement had already been reached), but it can also mean that the FVA-distance is not driven by the macromolecular biomass composition and the growth related parameters. Due to the use of measured and exact numbers, the model is more in line with the actual phenotype. Therefore, we think it is important to construct the model using correct data, as it is of meaning for future applications. Essential was that the minimal FVA-distance was kept relatively stable at the same range of numbers, even when the maximum FVA-distance significantly did decrease after restricting the flux for malate dehydrogenase. This shows that indeed possibly the best agreement was reached within limitations of constraint-based modeling. Given the fairly stable macro-molecular composition in the measured *μ* range it can be assumed that the flux distribution does not significantly change in *P. putida* KT2440 when the specific growth rate varies. This is relevant for the phenotype in case the strain is subjected to gene deletion. The experimental data used for validation encompass only a small part of the metabolic network. Consequently, the validation does not cover metabolic pathways outside the central metabolism.

The single mutant (PP1444) showed the highest increased synthesis of PHA when characterized by Poblete-Castro *et al*. [26]. Based on the FVA-analysis the *y*_*x/s*_^*max*^ was 0.32 g_DCW_·g_glc_^-1^. Poblete-Castro *et al.* reported a *Y*_*x/s*_^*max*^ of 0.25 g_DCW_ · g_glc_^-1^ for the single mutant, which seems a rather reasonable agreement. The remaining slight deviation might result from slight differences in the fermentation setup.

The contrast formed with transcriptomic data showed good agreement between the revalidated model and the experimental results. More than 90% of genes identified by the transcriptomics as potentially not expressed were involved in the catalysis of reactions not required for growth. For the reactions catalyzed by proteins of which zero-flux was predicted, only a limited number of genes were expressed. A lot of them are involved in the transport of amino acids, and the synthesis of co-factors, indicating that the bacterium tries to reduce the nutrient limitation. In general, with transcriptomic assays reproducible results are obtained [36]. Yet, the translation of the expression values into fluxes and *vice versa* needs to be done with care. For example, some genes might be expressed constitutively, consequently they do not always correspond with metabolic fluxes. Also there is no linear correspondence between the abundance of the mRNA transcript, as measured by transcriptomics, and the activity of the enzyme. This is due to a multitude of factors that influence the efficiency of protein production from mRNA as post-transcriptional regulation [37].

## Conclusions

By measuring carefully key biological parameters and contrasting simulation results with available experimental data, this work enabled to explain missing links and inconsistencies in the previously developed genome-scale metabolic model (iJP815), and improved considerably its accuracy, providing thereby a more solid basis for its use in designing biotechnological metabolic processes. Thus, the application of this approach under various conditions is a useful strategy to reconcile *in vivo* and *in silico* analysis and should therefore be incorporated into efficient strain and bioprocess optimization.

## Methods

### Strain and culture conditions

*P. putida* KT2440 (DSM6125) was aerobically grown in Biostat B bioreactors (Sartorius AG, Germany) at a working volume of 0.75 L, at 30°C, and pH 7 that was regulated with 1 M NaOH. The bacterium was grown on E-2 mineral medium (MM) [38] with 10 mM glucose. The bioreactor was sparged with air at 0.75 L · min^-1^. The stirring of the bioreactor was adjusted to 550 rpm, to ensure that the dissolved oxygen level was above 50% saturation. Antifoam 204 Sigma (an entirely organic anti-foaming agent, Sigma-Aldrich, USA) was added to the medium at a concentration of 0.02% (vol/vol). Fifteen bioreactor cultures were performed at various specific dilution rates (*D*) ranging from 0.05 to 0.64 h^-1^. All bioreactor cultures were started separately and were inoculated at 5% with a fresh pre-culture that was grown on a similar medium. After at least five residence times the desired specific growth rate (*μ*) was equal to *D*, at which time samples were taken.

### Analytical methods

The cell density of the cultures was determined by measuring the optical density (OD) of the culture against a water blank at 600 nm on a Eppendorf Biophotometer (Eppendorf AG, Germany). The dry cell weight concentration (*C*_*x*_) in g_DCW_ · L^-1^ was measured by filtering 10 mL of a culture over a MF mixed cellulose esters filter with 0.22 μm pores (Millipore, Ireland). Before usage the filters were dried at 80°C for 24 hours and weighted. After filtration, the filters were washed with 5 mL of 0.9% NaCl. Subsequently they were dried again at 80°C for 24 hours and weighted. Both OD_600_ and *C*_*x*_ measurements were done in triplicate for each *D*. To test whether the filter retracts any non-cell material such as salts and/or anti-foaming agent, the dry cell weight determination procedure was repeated for pure medium with anti-foaming agent and without carbon source. The measurements were repeated five times and yielded a residue of 0.356 (±0.023) g · L^-1^. This residue was subtracted from *C*_*x*_ measurements obtained in the previous step. Parallel, the OD_600_ and the *C*_*x*_ from 7 batch cultures of *P. putida* KT2440 in E-2 MM with 0 to 12.5 mM glucose were measured in triplicate. The following linear regression between the two measurements was determined: *C*_*x*_ = 0.35 · OD_600_, with a r^2^ of 1. Furthermore, in batch cultures it was shown that anti foam also has a significant positive influence at the measured cell density. The directly measured dry cell weight measurements from the bioreactor cultures showed a relation of 0.399 OD_600_. Therefore, the dry cell weight was compensated by multiplying with 0.35 divided by 0.399. The mean was taken from 2 to 3 *C*_*x*_-values measured by applying the OD_600_-*C*_*x*_ relationship as determined above. This was also done for 2 or 3 *C*_*x*_-values measured directly and corrected afterwards as described above. From these two means an average was taken. The off-gas was analyzed on CO_2_ content with the gas analyzer Servomex Xentra 4900 (Spectris, United Kingdom). The total amount of produced CO_2_ during each continuous bioreactor culture was calculated by analyzing the difference of its concentration in in- and outflowing gas and applying the gas law: *p · V* = *n · R · T*.

The cell viability was three times determined by live and dead cell discrimination assay using a standardized cell viability kit (BD Biosciences, USA). Before cell staining the cell count, the innercomplexity, and the size were determined once, which values were plotted with FlowJo software. The measurements were carried out using a FACSCalibur flow cytometer (BD Biosciences, USA). The liquid samples were beforehand diluted 100-fold in Tween 20-containing (0.01% w · v^-1^) phosphate buffered saline (PBS) (pH 7.4). The cell count was carried out at the flow of 60 μL · min^-1^.

To measure the glucose concentration (*C*_*glc*_) and ammonium concentrations, samples were taken from the feed medium and the supernatant sampled during the steady state of the bioreactor cultures. The samples were centrifuged at 16,873 · *g* for 2 min and filter sterilized (pore size of 0.2 μm), to preserve them and to remove solids and precipitated proteins, and stored at -20°C. The ammonium concentrations were measured in 15-fold diluted samples using the LCK 303 kit (Hach Lange, USA). According to the manufacturer the kit has the detection limit of 0.5 mg · L^-1^ and standard deviation of 0.3 mg · L^-1^. The *C*_*glc*_ was determined using a spectrophotometric enzyme assay kit (Roche, Germany), by measuring the absorbance at 340 nm in 10-fold diluted samples. According to the manufacturer the kit has the detection limit of 0.4 mg · L^-1^ and a standard deviation of 0.4-0.8 mg · L^-1^. The same samples were used to assess the presence of possible other metabolites in the efflux medium using nuclear magnetic resonance (NMR). This was performed in duplicate for the *D* of 0.26, 0.44 and 0.49 h^-1^. 1D ^1^H NMR spectra of aqueous supernatant containing 10% D_2_O or of supernatant containing a standard solution of sodium 3-(trimethylsilyl)propane-1-sulphonate dissolved in D_2_O to give a final volume of 0.66 mL were recorded on a Bruker AVANCE DMX600 NMR spectrometer at 300 K. The water signal was suppressed using standard Bruker software. For comparison purposes spectra of solutions of initial medium containing Antifoam 204 Sigma (Sigma-Aldrich, USA), glucose, sodium acetate and sodium 2-ketogluconate were recorded [39,40]. For quantitative analysis signals were referenced to the singlet signal of sodium 3-(trimethylsilyl)propane-1-sulphonate at a chemical shift of 0 ppm. In order to reach an appropriate signal to noise ratio, the spectra were recorded under standard conditions (sweep width: 20 ppm, acquisition time: 1.36 s, pulse delay: 1 s, number of scans: 1000, Bruker program noesypr1d). The detection limit for glucose was below 0.02 mM, for other metabolites below 0.1 mM.

To prepare samples for the measurement of RNA, carbohydrates, and lipids fractions, three medium samples of 5 mL each were taken and centrifuged at 1,557 · *g* at 4°C for 25 min. The supernatants were discarded and the pellets were stored in -70°C. Based on optical density two times the total concentration of RNA, carbohydrates and lipids in bacterial cells were determined according to the procedures as described by Benthin *et al.*[41], Herbert *et al*. [42] and Izard *et al*. [43], respectively.

For the analyses of the protein and DNA concentration in the biomass, one sample of 15 mL culture was taken and centrifuged at 20,201 · *g* at 4°C for 12 min. The pellet was washed with PBS (pH 7.4) and stored at -70°C. Prior to the analysis, the frozen cell pellet was re-suspended in 3 mL PBS and aliquoted in 3 equal-sized batches. One batch was used to determine the protein concentration in the cells. For this, the suspension was centrifuged at 16,873 · *g* at room temperature for 2 minutes and re-suspended in 200 μL protein extraction solution that consists per 10 mL re-swelling solution, of 46 mg 1,4-Dithiothreitol and a half tablet of protease inhibitor cocktail (Roche Diagnostics GmbH, Germany). Re-swelling solution consists of 84 g Urea (Mallinkrodt Baker, Inc., USA) dissolved in 200 mL distilled water, 10.0 g Serdolit (SERVA Electrophoresis GmbH, Germany), 30.4 g thio-urea (Sigma-Aldrich, USA), 8 g 3-[(3-Cholamidopropyl)dimethylammonio]-1-propansulfonat (Carl Roth GmbH, Germany), and 0.48 g tris base (Sigma-Aldrich, USA). After the re-suspension 2 μL lysozyme (100 mg · mL^-1^) was added and the cells were incubated at 37°C for 30 min while shaking at 400 rpm. During the incubation the samples were frozen three times with liquid nitrogen. Next, the incubation was continued at 37°C for 10 min while shaking at 1400 rpm. 50 μL of the supernatant was taken and centrifuged at 16,873 · *g* for 15 min at 4°C. The supernatant was diluted 50 times with re-swelling solution. Standards of bovine serum albumin of 0.5, 0.25, and 0.125 mg · mL^-1^ were prepared in triplicate. Three times 10 μL aliquots of the diluted supernatants as well as three separate samples of the three standards were mixed with 200 μL of fivefold diluted (with distilled water) Biorad protein assay solution (Bio-Rad laboratories GmbH, Germany). The absorbance of samples and standards was measured at 595 nm with a microplate reader (Bio-Rad model 3550-UV, USA). The absorbance values of the samples were recomputed to the concentrations using a regression line estimated from the absorbance of the standards. Additionally, the added lysozyme concentration was distracted from the total protein concentration.

The second batch of suspended cells was used to determine the concentrations of genomic DNA using the genomic DNA purification kit #K0512 (Fermentas, Canada). The procedure described by the manufacturer was followed until the step in which the DNA was dissolved in 100 μL of 1.2 M NaCl by gently vortexing. Then, 0.2 μL of 100 mg · mL^-1^ RNAse A (Qiagen, The Netherlands) was added, and the mixture was incubated at 37°C for 10 min under regular mixing by vortexing. To ensure the complete RNA degradation, the samples were analyzed with a formaldehyde agarose gel electrophoresis. The concentration of 20-fold diluted sample of DNA was measured at OD_260_ with an Eppendorf Biophotometer (Eppendorf AG, Germany). A total of 7 measurements were carried out of three dilutions, two of which were measured twice and one was measured three times.

From the continuous bioreactor culture with *P. putida* KT2440 at a *D* of 0.2 h^-1^ the amino acid composition of 1.5 mL medium was analyzed by an amino acid analyzer equipped with a ninhydrin detection system (Biochrom Ltd., UK) at Ansynth Service B.V. (The Netherlands). Two samples were centrifuged at 16,873 · *g* at room temperature for 2 min and the supernatant was discarded before sending the samples off.

### Growth parameters

To calculate the maintenance coefficient (*m*_*s*_) in g_glc_ · g_DCW_^-1^ · h^-1^ and the maximum biomass yield (*Y*_*x/s*_^*max*^) in g_DCW_ · g_glc_^-1^ of *P. putida* KT2440 the following formula of Pirt using the specific rate of substrate utilization (*q*_*s*_) in g_glc_ · g_DCW_^-1^ · h^-1^ was applied: *q*_*s*_ = *D · Y*_*x/s*_^*max-1*^ + *m*_*s*_[23]. The maximal specific growth rate (*μ*_*max*_) was determined by a wash out experiment. For this, the *D* was increased to 1.2 h^-1^ after cultivation at the *D* of 0.13 h^-1^ for five generation times. Within 3 hours the cell density was determined 10 times by measuring the OD_600_ of the culture. The *μ*_*max*_ was calculated by fitting the decrease of the cell density to the formula: *C*_*xt*_ *= C*_*x0*_ *· e*^*(*μ*-D)·t*^.

### Calculation of carbon and nitrogen-balance

To determine the carbon percentage in the biomass, the difference between carbon inflow (in form of glucose) and carbon outflow (in form of CO_2_) was calculated in g_carbon_ · L^-1^ · h^-1^. Dividing this value by the *R*_*x*_ (g_DCW_ · L^-1^ · h^-1^) yielded the carbon percentage in the biomass. To determine the nitrogen percentage of the biomass the difference between the nitrogen concentrations in the influx and efflux medium in g · L^-1^ was divided by the *C*_*x*_.

### Metabolic model

The metabolic model of *P. putida* model (iJP815) [8] was used. Several minor modifications that resulted from further development of the model were introduced. These are summarized (see Additional file 5).

### Creation of new biomass equation for *P. Putida* model (iJP815)

The new biomass equation for the iJP815 model was created based on a number of sources. First, the fraction of each type of macromolecule (Protein, DNA, RNA, Lipids, and Carbohydrates) was determined as described in Analytical methods. Since our protein determination method accounts only for water-soluble proteins, the fraction of proteins was increased by 12.2 percentage points – the amount of water-insoluble proteins. This value was assessed based on the fraction of 20% to 30% of proteins that diffuse into the membrane of *E. coli*[19,44]. Equivalence between water-insoluble proteins and membrane-bound proteins was assumed. Next, the composition of each type of macromolecule with regard to its building blocks (e.g. amino acids for proteins) was elucidated. The fractions were recomputed to represent the molar coefficients that are used in stoichiometric equations in the constraint-based models. The biomass equation was designed to produce 1 gram of biomass when the flux reaction equals to 1 and the fluxes are expressed in mmol · g_DCW_^-1^ · h^-1^.

#### Proteins

The amino acid composition was based on the experimental values obtained in continuous culture analyzed by Ansynth Service B.V. The amino acids that were not identified in this assay (Cysteine, Methionine, Tryptophan) were assumed to have identical molar fraction as in the *E. coli* biomass [10].

#### DNA

The fraction of each of four deoxyribonucleotide moieties was determined using the genomic sequence of the bacterium. Although replication forks of the genome are formed during growth it is assumed that the DNA sequence is equal for all cells.

#### RNA

The RNA composition was assumed to be identical to *E. coli* at a *D* of 0.6 h^-1^[28].

#### Lipids

The lipids were assumed to consist of two subclasses – lipopolysaccharide (LPS) and phospholipids. As the information regarding LPS was missing it was assumed that its fraction and molecular composition is the same as in *E. coli*[10]. The *in silico* LPS molecule in the constraint-based model consists only of lipid A and core oligosaccharide. The polysaccharide chains were not included, since their molecular composition and length are not well-defined, and, furthermore, they are accounted for in the carbohydrate biomass constituents. In addition, the cytidine diphosphate ethanolamine moiety was removed from the *in silico* LPS molecule as any evidence of the ability of *P. putida* KT2440 to synthesize this compound is missing. The composition of phospholipids was defined with regard to both head-groups and fatty-acid compositions. The composition of the former was identified from the measurements of the very close relative *Pseudomonas putida* S12 (phosphatidylethanoloamine – 73.7%, cardiolipin – 21.3%, phosphatidylglycerol – 4.9%; measured by Mylnefield Lipid Analysis, United Kingdom; Rita Volkers, personal communication), while the *in silico* fatty acid composition was based on experimental measurements of Stead [45].

#### Carbohydrates

The carbohydrate pool was assumed to be present entirely in the form of glycogen as a proxy for all sugar polymers.

In addition to these macromolecules peptidoglycan was assumed to be present [46] at a concentration of 8.6% (W·W^-1^). The biomass was augmented with vitamins and co-factors, albeit with several differences – the spermidine and UDP-Glucose were removed as there was no evidence that these two compounds can be produced by *P. putida* KT2440. As the small molecules are represented in minimal amounts in the biomass, the influence of their coefficients in the biomass equation on model predictions such as biomass yield (*Y*_*x/s*_) or flux-distribution is negligible. Important is only whether they are considered to be present or not as this influences, for example, the essentiality predictions for genes and reactions.

In order to evaluate the dependency between the fraction of every macromolecule and the growth rate, a regression analysis was performed. In addition, with a statistical F test the significance was determined if the slope differs from zero.

### Computation of maintenance coefficients

The GAM and NGAM are two coefficients that need to be fit in the model in order to make accurate flux predictions [11]. GAM – the amount of ATP that is dissipated when 1g_DCW_ is synthesized – was adjusted so that FBA-predicted (see below) *Y*_*x/s*_^*max*^ is equal to the experimentally measured value. NGAM – the amount of ATP that is dissipated for maintenance purposes by an amount of living cells (corresponding to 1g_DCW_) in a unit time (1 h) – was computed from the experimentally measured *m*_*s*_ by multiplying it by the maximal theoretical ATP yield (Y_ATP_) from glucose of 19.25 mol_ATP_ mol_glc_^-1^. The latter value was computed using the stoichiometry of the reactions and thus depends on the P:O ratio (1.33) assumed within the model.

### *In silico* flux predictions – FVA

Flux Variability Analysis (FVA) was used to predict flux values using the model. FVA is a variant of Flux Balance Analysis (FBA). FBA finds a flux distribution (values of fluxes of all reactions belonging to the model) that maximizes or minimizes a previously defined objective (usually *R*_*x*_ or substrate uptake rate) and calculates value of this objective given the constraints included into the model. This is achieved by means of linear optimization (maximization of a linear objective in constraint-based stoichiometric models poses a linear problem). In flux predictions reported here the objective was to minimize the glucose uptake rate of *P. putida* KT2440 while maintaining the specific μ at 0.2 h^-1^ (one of the *D* used in the continuous culture experiments). For more details on FBA, see Puchałka *et al.*, and Kauffman *et al.*[8,47]. Metabolic networks of living organisms are usually considerably underdetermined. This means that not all reaction fluxes can be uniquely determined by the FBA approach. FVA is a method that allows for rough top estimation of the allowed flux variability of each reaction, given by the FBA optimization. FVA computes for each reaction an interval of values inside of which the flux of the reaction can change without influencing the value of the objective function, provided that other fluxes are allowed to vary freely within their constraints [48-50]. For each FVA-simulation the sums of the minimal and maximal distances provide two distance values (minimal and maximal). This pair of values will be called a “FVA-distance” and is calculated as square distance.

### Comparison of the model output with ^13^C flux measurement data

The FVA-predictions were compared with ^13^C flux measurements of Fuhrer *et al*. [16]. In these measurements fluxes of the reactions belonging to central metabolic pathways (Pentose phosphate Pathway, Entner-Doudoroff Pathway and TCA Cycle) were determined for *P. putida* KT2440 grown in Erlenmeyer flasks in minimal medium with glucose. To assess the agreement between the experimental measurements and FVA-predictions, the minimal and maximal FVA-results were compared to the experimental data.

### Comparison of model predictions with transcriptomic data

The transcriptomic data of van Duuren *et al*., [17] were used as one of the sources for model validation. This was done by comparing pathways, using gDNA as a template, with the expression levels of RNA. The relative expression analysis was carried out for *P. putida* continuously grown under solely glucose limitation at *D* 0.2 h^-1^. Four microarray experiments were used for the validation. These included the mutant *P. putida* KT2440-JD1 and the WT strain. The inclusion of the mutant strain was prompted by the lack of significant differences in expression of genes involved in the central metabolism when grown on glucose. The genome of the mutant comprises a mutation in gene *catR*, the regulator of the *cat* operon involved in the benzoate pathway. Several methods were used to estimate the correlation between the transcriptomic data and the model predictions. These are described in the result section of the manuscript.

## Abbreviations

MM: Mineral medium; PBS: Phosphate buffered saline; LPS: Lipo poly sacchariden; OD: Optical density; μ: Specific growth rate; μmax: Maximum specific growth rate; D: Specific dilution rate; Cx: Dry cell weight concentration; Cglc: Glucose concentration; qs: Specific rate of substrate utilization; ms: Maintenance coefficient; Yx/s: Biomass yield; Yx/smax: Maximal biomass yield; Rglc: Glucose uptake rate; Rx: Biomass production rate; RCO2: CO_2_ production rate; GAM: Growth associated maintenance; NGAM: Non-growth associated maintenance; NMR: Nuclear magnetic resource; YATP: ATP yield; FVA: Flux variability analysis; FBA: Flux balance analysis.

## Competing interests

The authors declare that they have no competing interests.

## Authors’ contributions

JvD performed the lab experiments. JP and RB performed the computational experiments. JvD and JP wrote together the manuscript. AM, CW, GE, and VdS were responsible for the drafting and editing. All authors read and approved the final manuscript.

## Supplementary Material

Additional file 1: Figure S1 The dry cell weight concentration (*Cx*) (♦), glucose concentration (*C*_*glc*_) (■) (g∙L^-1^) and CO_2_ (▲) (g · L^-1^ · h^-1^) of *P. putida* KT2440 at various dilution rates (*D*) (h^-1^) on MM with 10 mM glucose. Click here for file

Additional file 2: Figure S2 A/B Median of the forward scatter (FSC) (♦) and side scatter (SSC) (■) as well as the cell counting (mL^-1^) (▲) at various specific growth rates (*μ*) (h^-1^). Click here for file

Additional file 3: Table S1 Genes predicted essential by the iJP815 model for which transcriptomics data suggest a lack of expression. Colors mark distinctive groups discussed in the text. Click here for file

Additional file 4: Table S2 Genes predicted to be involved in the catalysis of reactions with predicted zero flux, for which the transcriptomics data suggest high expression. Colors mark distinctive groups discussed in the text. Click here for file

Additional file 5 Modifications to the original iJP815 model. Click here for file
