# Supplementary figures and images for: Reconciling in vivo and in silico key biological parameters of Pseudomonas putida KT2440 during growth on glucose under carbon-limited condition
Source: BMC Biotechnol. 2013 Oct 29;13:93. doi: 10.1186/1472-6750-13-93 (PMC3829105; doi:10.1186/1472-6750-13-93)

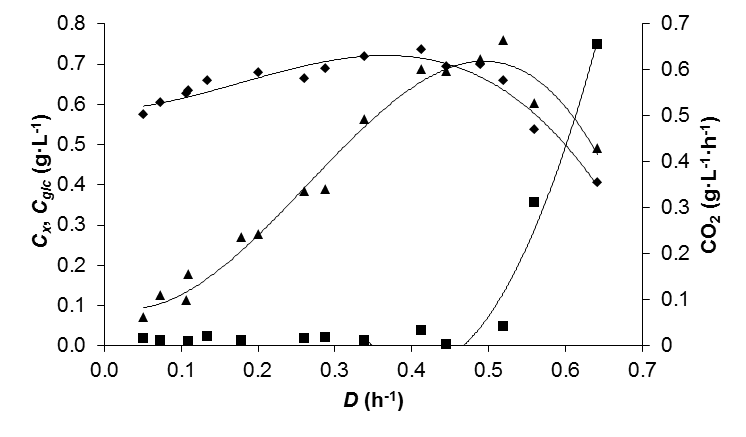

Supplement: Additional file 1: Figure S1 — The dry cell weight concentration (Cx) (♦), glucose concentration (C glc ) (■) (g∙L-1) and CO2 (▲) (g · L-1 · h-1) of P. putida KT2440 at various dilution rates (D) (h-1) on MM with 10 mM glucose. [file 1472-6750-13-93-S1.docx]

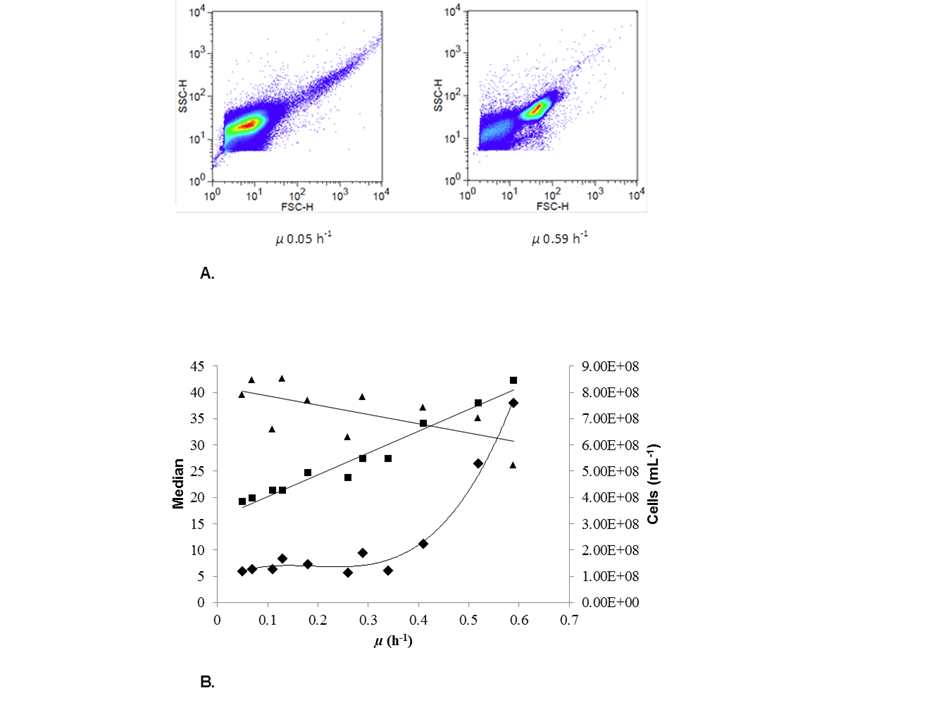

Supplement: Additional file 2: Figure S2 — A/B Median of the forward scatter (FSC) (♦) and side scatter (SSC) (■) as well as the cell counting (mL-1) (▲) at various specific growth rates (μ) (h-1). [file 1472-6750-13-93-S2.docx]

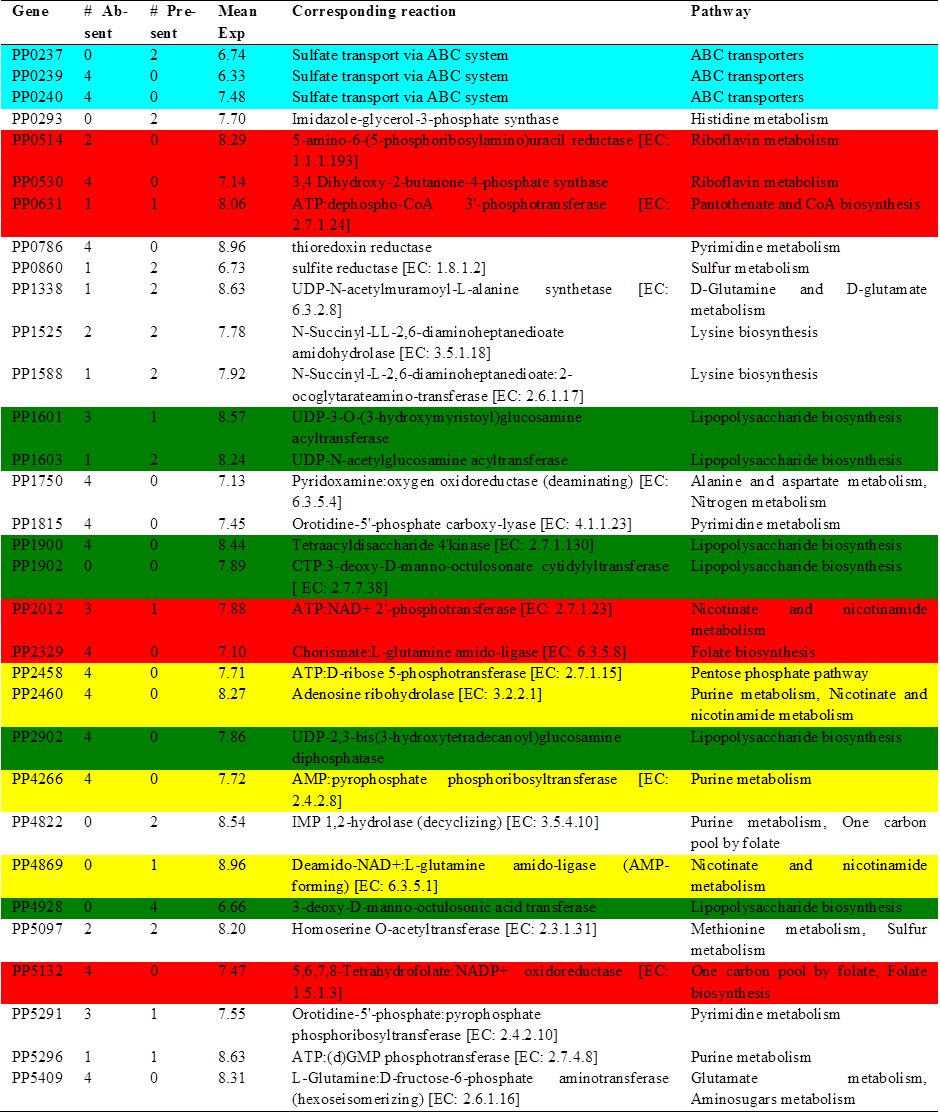

Supplement: Additional file 3: Table S1 — Genes predicted essential by the iJP815 model for which transcriptomics data suggest a lack of expression. Colors mark distinctive groups discussed in the text. [file 1472-6750-13-93-S3.docx]

**
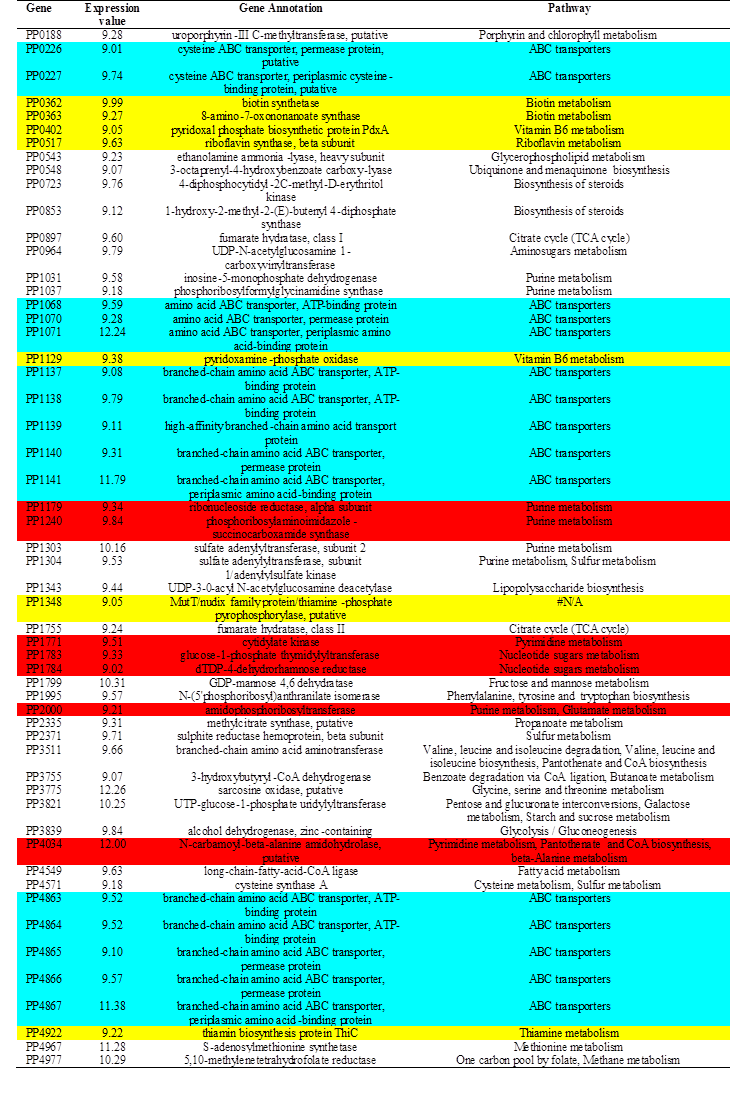
**

Supplement: Additional file 4: Table S2 — Genes predicted to be involved in the catalysis of reactions with predicted zero flux, for which the transcriptomics data suggest high expression. Colors mark distinctive groups discussed in the text. [file 1472-6750-13-93-S4.docx]
