# Supplementary material for: Reconciling in vivo and in silico key biological parameters of Pseudomonas putida KT2440 during growth on glucose under carbon-limited condition
Source: BMC Biotechnol. 2013 Oct 29;13:93. doi: 10.1186/1472-6750-13-93 (PMC3829105; doi:10.1186/1472-6750-13-93)
Supplement: Additional file 5 — Modifications to the original iJP815 model. [file 1472-6750-13-93-S5.docx]

1. Addition of the following reactions responsible for the synthesis of fatty acids which the original biomass equation did not account for:
   1. S-2-hydroxylauroyl-ACP synthase:
      (2) Dodecanoyl-ACP (n-C12:0ACP) + Oxygen --> (2) 2-hydroxydodecanoyl-[acyl-carrier protein]
   2. cis-9,10-methylene hexadecanoyl-acp synthase:
      Hexadecenoyl-ACP (n-C16:1ACP) + S-Adenosyl-L-methionine --> S-Adenosyl-L-homocysteine + cis-9,10-methylene hexadecanoyl-acp
2. Modification of the stoichiometry of the phosphatidate synthase reaction to account for the new composition of the acyl residues in phospholipids:
   *old:* (2) Myristoyl-ACP (n-C14:0ACP) + (7) cis-9-hexadecenoyl-acp + (5) cis-7-tetradecenoyl-acp + (36) Palmitoyl-ACP (n-C16:0ACP) + (50) sn-Glycerol 3-phosphate + (50) cis-11-octadecenoyl-acp --> (100) acyl carrier protein + phosphatidate (E.coli) **
   *new:* (7) (R)-3-Hydroxydecanoyl-[acyl-carrier protein] + (26) cis-9-hexadecenoyl-acp + (6) cis-9,10-methylene hexadecanoyl-acp + (50) sn-Glycerol 3-phosphate + (3) Dodecanoyl-ACP (n-C12:0ACP) + (6) (R)-3-Hydroxydodecanoyl-[acyl-carrier protein] + (29) Palmitoyl-ACP (n-C16:0ACP) + (6) 2-hydroxydodecanoyl-[acyl-carrier protein] + (17) cis-11-octadecenoyl-acp --> Phosphatidic acid (PPU) + (100) acyl carrier protein
3. Modification of the elemental composition of the following phospholipid compounds to account for the new composition of the acyl residues in phospholipids:
   1. phosphatidate, old: C1836H3398O400P50, new: C1682H3116O413P50
   2. phosphatidylglycerol, old: C1986H3748O500P50, new: C1832H3466O513P50
   3. phosphatidylserine, old: C1986H3698N50O500P50, new: C1832H3416N50O513P50
   4. CDP-diacylglycerol, old: C2286H3998N150O750P100, new: C2132H3716N150O763P100
   5. cardiolipin, old: C3822H7096O850P100, new: C3514H6532O876P100
   6. phosphatidylglycerol phosphate, old: C1986H3698O650P100, new: C1832H3416O663P100
4. Substitution of multi-step mechanism of the following enzymes with a single-step lumped reaction:
   1. Pyruvate dehydrogenase
   2. 2-oxoglutarate dehydrogenase
   3. 2-oxoadipate dehydrogenase
   4. Acetolactate synthase
   5. (S)-2-Aceto-2-hydroxybutanoate synthase

This change was made to separate better the fluxes between these multi-step reactions, as some of the sub-steps overlap between them. This influences the computation of the FVA-distances. Since all these reactions are catalyzed by enzyme complexes, it can be assumed that the intermediates are not exchanged between different reactions and the approximation by lumped reactions is a correct one.
